# Cobalt-centred boron molecular drums with the highest coordination number in the CoB_16_^−^ cluster

**DOI:** 10.1038/ncomms9654

**Published:** 2015-10-12

**Authors:** Ivan A. Popov, Tian Jian, Gary V. Lopez, Alexander I. Boldyrev, Lai-Sheng Wang

**Affiliations:** 1 Department of Chemistry and Biochemistry, Utah State University, Logan, Utah 84322, USA; 2 Department of Chemistry, Brown University, Providence, Rhode Island 02912, USA

## Abstract

The electron deficiency and strong bonding capacity of boron have led to a vast variety of molecular structures in chemistry and materials science. Here we report the observation of highly symmetric cobalt-centered boron drum-like structures of CoB_16_^−^, characterized by photoelectron spectroscopy and *ab initio* calculations. The photoelectron spectra display a relatively simple spectral pattern, suggesting a high symmetry structure. Two nearly degenerate isomers with D_8d_ (**I**) and C_4v_ (**II**) symmetries are found computationally to compete for the global minimum. These drum-like structures consist of two B_8_ rings sandwiching a cobalt atom, which has the highest coordination number known heretofore in chemistry. We show that doping of boron clusters with a transition metal atom induces an earlier two-dimensional to three-dimensional structural transition. The CoB_16_^−^ cluster is tested as a building block in a triple-decker sandwich, suggesting a promising route for its realization in the solid state.

## Abstract (web summary)

Boron is known to form a wide variety of molecular structures. Here, the authors observe the highly symmetric cobalt-centered boron drum-like structure of CoB_16_^−^, characterized by photoelectron spectroscopy and ab initio calculations, in which the cobalt atom is sixteen-coordinate.

## Graphical abstract

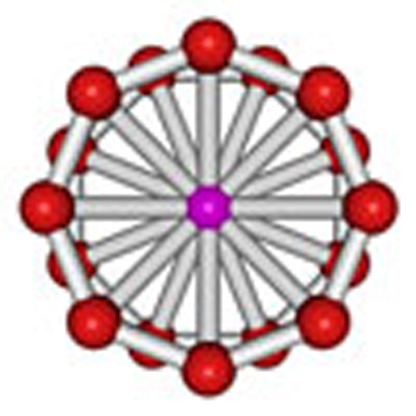

Boron, the fifth element in the periodic table, possesses such diverse chemical structures and bonding that are second only to carbon. Bulk boron consists of connected three-dimensional (3D) cages in many of its allotropes^1^,^2^ and boron-rich borides^3^,^4^. However, for isolated clusters it was computationally shown^5^,^6^ that icosahedral cage structures of B_12_ and B_13_ were unstable, even though they were initially proposed as possible candidates for these two clusters^7^. Over the past decade, small anionic boron clusters have been systematically characterized both experimentally and theoretically to exhibit planar or quasi-planar structures in their ground states up to B_27_^−^ (refs 8, 9, 10). Recent works show that anionic boron clusters continue to be two-dimensional (2D) at B_30_^−^ (ref. 11), B_35_^−^ (ref. 12) and B_36_^−^ (ref. 13). The 2D-to-3D transition was suggested to occur at B_20_ for neutral^14^, and at B_16_^+^ for cationic clusters^15^. Very recently it is shown that the transition from 2D to fullerene-like 3D structures occurs in negatively charged boron clusters at about 40 boron atoms in B_39_^−^ (ref. 16) and B_40_^−^ (ref. 17). Due to the nearly spherical shapes of these clusters, they have been named borospherenes. Doping boron clusters with a single metal atom opens a new avenue to create clusters with novel structures and chemical bonding. It has been experimentally observed that various transition metal atoms can be placed inside of monocyclic boron rings to form beautiful molecular wheel-type structures (M©B_*n*_^−^)^18^, following an electronic design principle inspired by the doubly σ and π aromatic B_9_^−^ cluster^19^. It was shown that the Nb©B_10_^−^ and Ta©B_10_^−^ clusters possess the record coordination number of 10 in the planar environment for the central metal atom^20^. These clusters have pushed the limits of structural chemistry.

Here we report the observation of a large metal-doped boron cluster of CoB_16_^−^, which is produced using a laser vaporization cluster source and characterized by photoelectron spectroscopy (PES). Extensive computational searches reveal that there are two nearly degenerate structures for CoB_16_^−^, which are indistinguishable at the highest level of theory employed. They both possess tubular double-ring framework and give similar photoelectron spectral patterns. The structures can be viewed as two B_8_ rings sandwiching a Co atom, reminiscent of a drum and giving rise to the highest coordination number known in chemistry thus far.

## Results

### Experimental results

**Figure 1:**
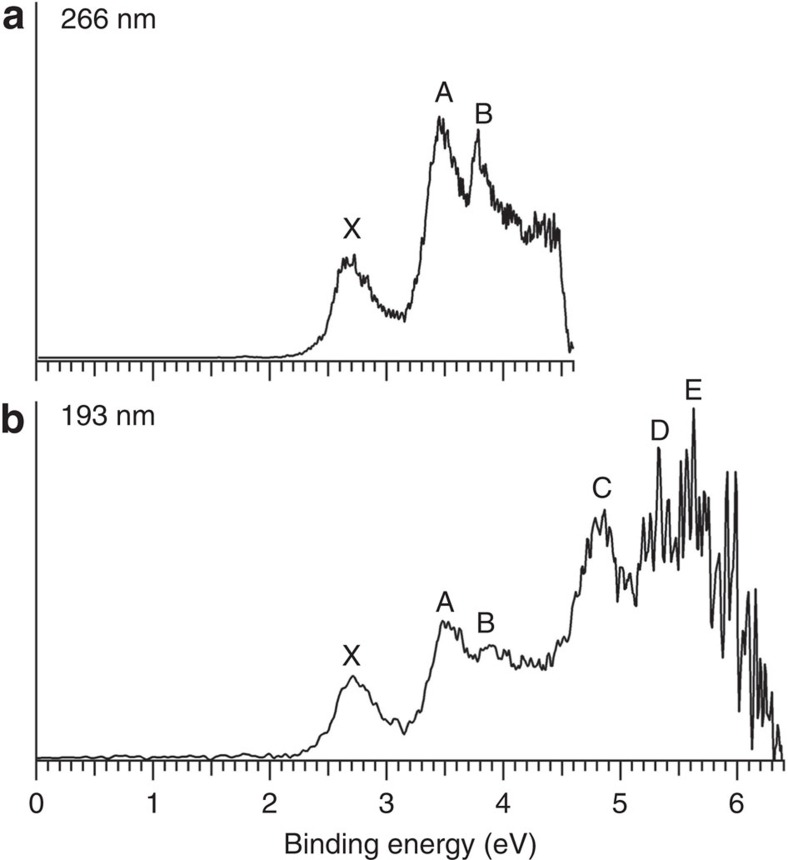
Photoelectron spectra. Photoelectron spectra (**a**) at 266 nm (4.661 eV) and (**b**) at 193 nm (6.424 eV) of CoB_16_^−^.

The photoelectron spectra of CoB_16_^−^ at two photon energies are displayed in Fig. 1. The lowest binding energy band (X) represents the electron detachment transition from the anionic ground state to that of neutral CoB_16_. The higher binding energy bands, A, B, …, denote detachment transitions to the excited states of neutral CoB_16_. The vertical detachment energies (VDEs) for all observed bands are given in Table 1, where they are compared with the calculated VDEs.

The 266 nm spectrum (Fig. 1a) reveals three well-resolved PES bands for CoB_16_^−^. The band X gives rise to a VDE of 2.71 eV. The adiabatic detachment energy (ADE) for band X was evaluated from its onset to be 2.48 eV, which also represents the electron affinity of neutral CoB_16_. The width of band X suggests an appreciable geometry change between the ground electronic state of CoB_16_^−^ and the ground electronic state of CoB_16_. Following a relatively large energy gap, an intense and broad band A is observed at a VDE of 3.45 eV and a close-lying band B at a VDE of 3.78 eV. The 193 nm spectrum (Fig. 1b) shows nearly continuous signals beyond 4 eV. The sharp spikes above 5 eV in the high binding energy side of the 193 nm spectrum are due to statistical noises because of low electron counts. An intense and broad band C is clearly observed at a VDE of 4.86 eV. Two more bands can be tentatively identified at higher binding energies, D (VDE: ∼5.3 eV) and E (VDE: ∼5.6 eV). Overall, the PES spectral pattern is relatively simple, suggesting that the framework of the CoB_16_^−^ cluster is likely to have high symmetry.

### Theoretical results and comparison with experiment

**Figure 2:**
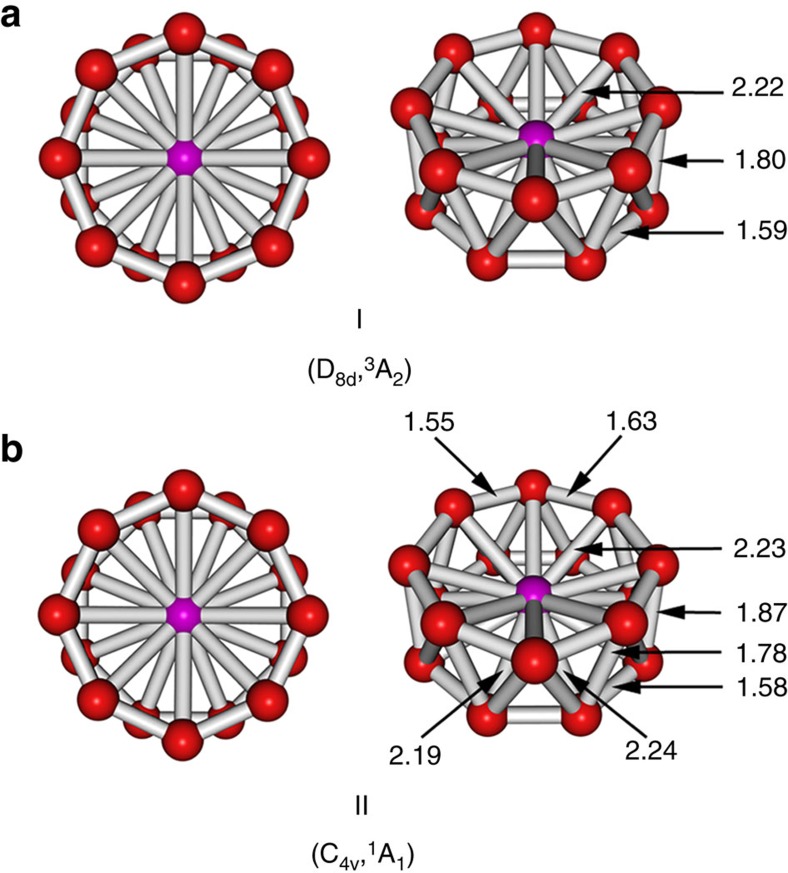
Two views of isomer I and isomer II of the CoB_16_^−^ cluster. The point group symmetries and spectroscopic states of isomer I (**a**) and isomer II (**b**) are shown in parentheses. Sticks drawn between atoms help visualization and do not necessarily represent classical 2c–2e B–B or Co–B bonds here and elsewhere. All distances are in Å.

Extensive structural searches were initially done at the PBE0/3-21G level of theory with the follow-up calculations (Δ=25 kcal mol^−1^) at the PBE0/Def2-TZVP level of theory, which led to two similar drum-like structures: isomer **I** (D_8d_, ^3^A_2_) and isomer **II** (C_4v_, ^1^A_1_) identified as the global minima for CoB_16_^−^ (Fig. 2). These two highly symmetric structures, consisting of a central Co atom sandwiched by two B_8_ monocyclic rings, are found to be almost degenerate at various levels of theory (Supplementary Fig. 1 and Supplementary Table 1). Clearly, the method dependency of predicting relative energies of the low-lying structures for CoB_16_^−^ suggests the importance of comparison with experiment in determining the global minimum. We previously studied how optimized geometries of small boron clusters differed at density functional theory (DFT) and CCSD(T) levels of theory^21^,^22^. We found that B3LYP/6-311+G* geometries are quite close (within 0.03 Å between nearest boron atoms) to those at the CCSD(T)/6-311+G* level of theory. We also compared the geometries of boron clusters at PBE0/6-311+G* and B3LYP/6-311+G*, and found that they are also very close^23^. Therefore, PBE0/3-21G level of theory was used for the preliminary search and PBE0/Def2-TZVP for the final optimized geometries of CoB_16_^−^. The highest level of theory employed (ROCCSD(T)/6-311+G(2df)//PBE0/Def2-TZVP (this abbreviation means that single-point energy calculations were performed at ROCCSD(T)/6-311+G(2df) using optimized UPBE0/Def2-TZVP geometries here and elsewhere) indicates 1.4 kcal mol^−1^ energy difference including zero-point energy corrections (Supplementary Fig. 1). This small value is in the range of the theoretical errors for such a complex transition-metal-doped boron cluster. Therefore, isomers **I** and **II** should be considered to be degenerate based on our calculations. Figure 2 shows the small differences in bond distances between isomers **I** and **II**; the latter is not significantly distorted from the D_8d_ symmetry. The B–B bond lengths of the B_8_ rings for both isomers are in the range of 1.55–1.63 Å, similar to the corresponding values (1.56 Å) in the Co©B_8_^−^ molecular wheel^18^. The nearest isomer **III** (C_2_, ^1^A) is 8.7 kcal mol^−1^ higher in energy at the ROCCSD(T) method and represents a distorted drum-like structure composed of two B_7_ rings with two B atoms outside the drum (Supplementary Fig. 1). In fact, the majority of the low-lying isomers within 20 kcal mol^−1^ (Supplementary Fig. 1) represent various derivatives (drum-like or possessing principal geometrical features of the drum-like structure) of isomers **I** and **II**, showing the stability of the drum-like structures. It should be noted that there are significant bonding interactions between the two B_8_ rings and between the Co atom and all 16 B atoms in both isomers **I** and **II** (*vide infra*). Interestingly, the drum structure in a quintet state (isomer **XIV** in Supplementary Fig. 1) appears to be the most stable one out of all other quintet isomers. It should be mentioned that there were two previous DFT calculations on similar drum-like structures of neutral boron clusters doped with transition metal atoms^24^,^25^.

To facilitate comparisons between the experimental and theoretical results, we calculated low-lying VDEs of isomers **I** and **II** of CoB_16_^−^ using three methods (Table 1). We found that the VDEs computed using the two DFT methods are not very impressive; but we observed good agreement between the theoretical VDEs at ROCCSD(T)/6-311+G(2df) and the experimental data for the first two detachment channels (Table 1). Since isomer **I** is open shell, the electron detachment energy from the doubly degenerate 4e_2_-HOMO should lead to a doublet final state for the neutral. The computed VDE at ROCCSD(T) is 2.59 eV, compared with the experimental VDE of 2.71 eV. The next electron detachment from the non-degenerate 2b_2_-HOMO-1 should lead to both a quartet and a doublet final state, with the quartet being lower in energy. The calculated VDE for the quartet final state at ROCCSD(T) is 3.28 eV, compared with the VDE of the A band at 3.45 eV. Unfortunately, we were not able to calculate any higher VDEs because of the limitation of the ROCCSD(T) method. However, we believe that the good agreement between experiment and theory for the first two VDEs provides sufficient credence for the identified drum-like isomer **I** for the CoB_16_^−^ cluster.

Isomer **II** gives very similar theoretical VDEs as isomer **I** at all three levels of theory, consistent with the similarities in their geometries. Since isomer **II** is a closed shell species, we were able to calculate only the first VDE value at the ROCCSD(T) method as 2.61 eV, also in good agreement with the experimental data. Furthermore, the calculated ADEs of isomer **I** (2.45 eV) and isomer **II** (2.43 eV) (PBE0/Def2-TZVP) are in excellent agreement with the experimentally measured ADE value of 2.48 eV. We should point out that there is a Jahn–Teller distortion for the neutral CoB_16_ drum-like structure of isomer **I**, consistent with the broad X band observed in the PES spectra (Fig. 1). Indeed, the calculated relaxed neutral CoB_16_ structure **I**^**0**^ (Supplementary Fig. 2 and Supplementary Table 1) has lower symmetry (C_2v_), as one would expect for the Jahn–Teller distorted structure due to the occupation of the doubly degenerate HOMO (4e_2_) of isomer **I** by a single electron. In fact, the HOMO (4b_2_) of isomer **II** originates from the HOMO (4e_2_) of isomer **I** when one of the doubly degenerate orbitals is doubly occupied. Therefore, the detachment of one electron from the doubly occupied HOMO (4b_2_) of isomer **II** leads to the same neutral structure **I**^**0**^. The high relative energy of isomer **III**, as well as its appreciably higher theoretical first VDE of 3.65 eV (Supplementary Table 2), makes this cluster unlikely to be populated in the molecular beam in any appreciable amount.

## Discussion

Tubular (or drum-like) boron clusters have been of interest for many years, because they can be considered as the embryos for boron nanotubes^14^. However, such drum-like structures have never been observed experimentally for bare boron clusters, even though they have been shown to be stable computationally^14^,^26^,^27^,^28^,^29^. For instance, the B_20_ cluster was first suggested as the global minimum on the basis of theoretical calculations^14^, but it has not been observed or confirmed experimentally^29^. Tubular structures were also studied for the bare B_16_^+^, B_16_, B_16_^−^ and B_16_^2−^ species^15^,^30^. For the B_16_^+^ cationic cluster, the tubular structure was suggested to be the global minimum^15^, whereas the tubular structures of both B_16_ and B_16_^−^ were found to be high-energy isomers^30^. Clearly, the strong coordination interactions with the Co atom significantly stabilize the tubular B_16_ to give the drum-like global minima (structures **I** and **II**) for CoB_16_^−^. Bare anionic boron clusters are found to be 2D up to B_36_^−^ (ref. 13), while some transition-metal-doped anionic boron clusters are found to preserve the planar boron framework on metal doping^31^,^32^. The largest experimentally observed metal-doped boron cluster (CoB_12_^−^) maintains a similar planar geometry for the B_12_ moiety^32^. Hence, the doping of the Co atom induces an earlier 2D-to-3D transition for boron clusters, as shown by the 3D isomers **I** and **II** of CoB_16_^−^. In fact, the CoB_16_^−^ drum structure represents the highest coordination number known in chemistry today. The previous highest coordination number known experimentally was 15 for [Th(H_3_BNMe_2_BH_3_)_4_] (ref. 33), though theoretical studies have suggested the highest coordination numbers of 15 in PbHe_15_^2+^ (ref. 34) and 16 in the Friauf–Laves phases in MgZn_2_ or MgNi_2_ (ref. 35). Endohedral fullerenes (M@C_60_) have been observed^36^,^37^, but the metal atom in those cases interacts with the C_60_ shell primarily ionically and it does not stay in the centre of C_60_.

It is interesting to point out that the B–B distances in the B_8_ rings of both isomers **I** and **II** of CoB_16_^−^ and the bare tubular B_16_ are very similar (Supplementary Table 3). To gain insight into the chemical bonding of the CoB_16_^−^ drums, we performed chemical bonding analyses for isomers **I** and **II** using the Adaptive Natural Density Partitioning (AdNDP) method^38^, which is an extension of the popular Natural Bond Orbital method^39^. It should be noted that the bonding in some double-ring tubular boron clusters has been discussed previously^9^,^40^,^41^.

**Figure 3:**
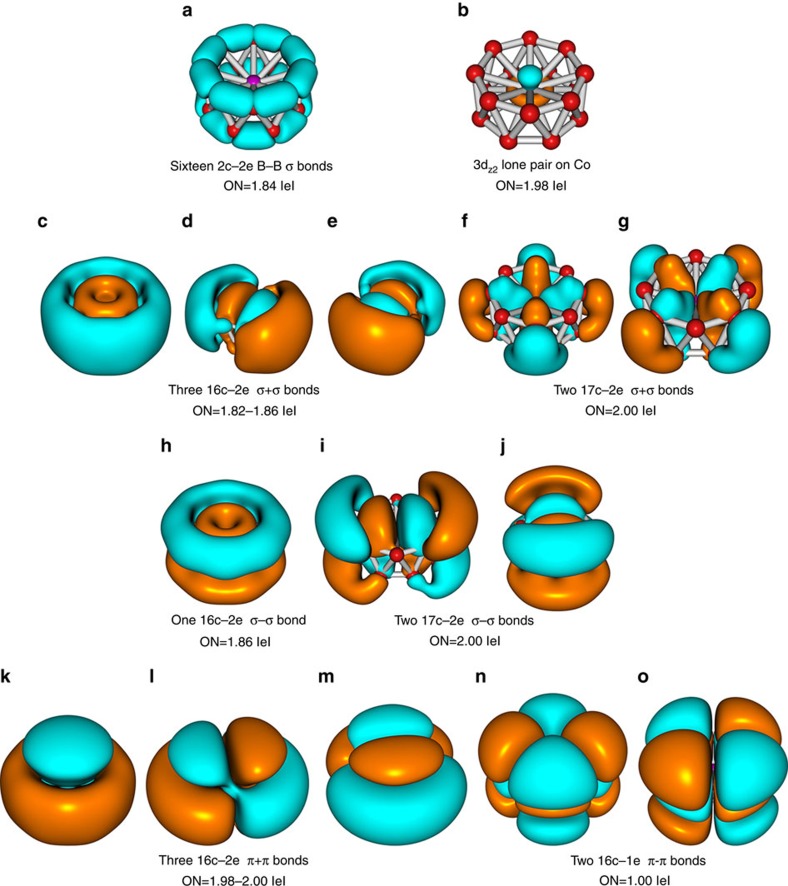
Chemical bonding picture. (**a**–**o**) The overall chemical bonding picture (**a**–**o**) obtained for the isomer I of the CoB_16_^−^ molecular drum via the UAdNDP analysis. ON denotes occupation number here and elsewhere.

Since isomer **I** has two unpaired electrons, we used the unrestricted AdNDP (UAdNDP) analysis, which enables treatments of the α and β electrons separately. To obtain an averaged result for a bond (Fig. 3), we added the UAdNDP results for the α and β electrons of the same type of bonds. According to the UAdNDP analysis results, the 58 valence electrons in CoB_16_^−^ can be divided into four sets. The first set (Fig. 3a,b) consists of localized bonding elements, while the other three sets (Fig. 3c–g, h–j, k–o) are composed of delocalized bonding elements. In the first set, the UAdNDP analysis for isomer **I** revealed the following localized bonding elements: one lone pair (1c–2e bond) (Fig. 3b) of 3d_z_2-type on Co with an occupation number (ON) of 1.98 |e| and sixteen 2c–2e B–B σ-bonds (Fig. 3a) with ON=1.84 |e| within each B_8_ ring (all superimposed onto the B_16_ fragment in Fig. 3), which can also be viewed as 3c–2e bonds with the ON=1.96 |e| responsible for the bonding between the boron rings. In the last case, a boron atom from the neighbouring ring contributes somewhat (0.12 |e|) to the formation of the 3c–2e σ-bond. The 2c–2e B–B σ-bonds are very similar to the peripheral B–B bonds found in all 2D boron clusters^8^,^9^,^10^. The second set includes five delocalized σ bonds (denoted as σ+σ), which are formed from delocalized σ bonds between the two B_8_ rings. Since the σ orbitals between the two boron rings overlap positively, we designate them as σ+σ in the *second* set, which constitutes σ-aromaticity according to the 4*n*+2 (*n*=2) Hückel rule. The three delocalized 16c–2e σ+σ bonds (Fig. 3c–e) with ON=1.82–1.86 |e| involve only σ-bonding within the boron rings, whereas the two delocalized 17c–2e σ+σ bonds (Fig. 3f,g) come primarily from the 3d_xy_ and 3d_x_2_–y_2 AOs of Co interacting with the boron rings. It should be noted that the direct covalent interactions between Co and the B_16_ unit via the 3d_xy_ and 3d_x_2_-y_2 AOs of Co are found to be around 0.6 |e| according to the AdNDP analysis. The third set (Fig. 3h–j) shows three delocalized σ–σ bonds, which represent bonding interactions within each ring, but anti-bonding interactions between the two boron rings. This set of delocalized bonds also constitutes σ-aromaticity according to the 4*n*+2 (*n*=1) Hückel rule. In the *third* set, the 16c–2e σ–σ bond (Fig. 3h) involves mainly the two boron rings, whereas the two 17c–2e σ–σ bonds (Fig. 3i,j) involve interactions between the 3d_xz_ and 3d_yz_ AOs of Co with the boron rings. The direct covalent interaction of the 3d_xz_ and 3d_yz_ AOs of Co with the boron kernel is assessed to be around 0.5 |e|. The *last* set includes five delocalized bonds, which represent π–π interactions between the boron rings: three 16c–2e π–π bonds (Fig. 3k–m) with ON=1.98–2.00 |e| and two 16c–1e π–π bonds (Fig. 3n,o) with ON=1.00 |e| (one unpaired electron on each bond). The eight π electrons in the *last* set suggest π-aromaticity according to the 4*n* rule (*n*=2) for triplet states. Therefore, the stability of isomer **I** of CoB_16_^−^ can be considered to be due to the double σ- and π-aromaticity and bonding interactions of the 3d AOs of Co with the B_8_ rings.

As expected, isomer **II** of CoB_16_^−^, which is close in energy and geometry to isomer **I**, has almost the same bonding pattern as that of isomer **I** (Supplementary Fig. 3). All the bonding elements found in isomer **I** are also found in isomer **II** except for the *last* set (Supplementary Fig. 3k–n). Since isomer **II** is closed shell, eight electrons in the *last* set are observed to form four 16c–e π–π bonds with ON=1.98–2.00 |e|, rendering this isomer π-antiaromatic. Hence, isomer **II** exhibits conflicting aromaticity (σ-aromatic and π-antiaromatic), which leads to some distortion to C_4v_ symmetry compared to the D_8d_ symmetry of the doubly aromatic isomer **I**. As was mentioned earlier, the HOMO (4b_2_) of isomer **II** originates from the HOMO (4e_2_) of isomer **I** when one of the doubly degenerate orbitals is doubly occupied. Indeed, occupation of only one degenerate MO by two electrons causes the electronic instability, which causes the geometric rearrangement of isomer **II** lowering the D_8d_ symmetry to C_4v_.

To understand the interactions between Co and the tubular B_16_ host, we have performed AdNDP analyses for the neutral B_16_ tubular isomer (Supplementary Fig. 4). Similar to isomers **I** and **II** of CoB_16_^−^, the AdNDP analyses give 16 2c–2e B–B σ-bonds with ON values of 1.70 |e| within the two B_8_ rings. The encapsulation of Co strengthens the B–B σ-bonds within each B_8_ ring in CoB_16_^−^, but weakens the inter-ring interactions, compared with the bare B_16_, as reflected by their ON values (Fig. 3 and Supplementary Fig. 4) and the B–B bond lengths (Supplementary Table 3). The remaining 16 electrons in B_16_ participate in delocalized bonding: five 16c–2e σ+σ bonds and three 16c–2e π–π bonds, rendering the tubular B_16_ doubly σ- and π-aromatic. The major difference in chemical bonding between the drum-like B_16_ and CoB_16_^−^ comes from two factors: (1) the formation of an additional set (Fig. 3h–j) of the delocalized σ–σ bonds in CoB_16_^−^; and (2) participation of Co 3d AOs in the two 17c–2e σ+σ bonds (Fig. 3f,g). Both factors are consistent with structural changes between CoB_16_^−^ and B_16_. There are strong bonding interactions between Co and the B_16_ host in CoB_16_^−^ to stabilize the tubular B_16_ structure, because the global minimum of B_16_ is planar^30^.

Isomer **I** of CoB_16_^−^ is open shell with two unpaired electrons, whereas isomer **II** can be viewed as a result of Jahn–Teller distortion from isomer **I**. Addition of two electrons to isomers **I** or **II** would create a closed shell and doubly aromatic CoB_16_^3−^ species with D_8d_ symmetry. Our calculations indeed confirmed this hypothesis: CoB_16_^3−^ was found to be a minimum on the potential energy surface with very similar bond distances as in isomer **I** (Supplementary Table 3). The triply charged CoB_16_^3−^ species can be electronically stabilized by external akali metal cations, such as in Na_2_CoB_16_^−^. Since ligation would be needed to ultimately synthesize CoB_16_^−^, we considered a triple-decked [CoB_16_(CaCp)_2_]^−^ sandwich complex (Supplementary Fig. 5), using the divalent Ca atoms and the aromatic C_5_H_5_^−^ (Cp^−^) ligands. It should be mentioned that similar [CpLiB_6_LiCp]^2−^ triple-decked complex^42^ with the double antiaromatic B_6_^2−^ unit was previously suggested to be stable and viable experimentally. We found that the [CoB_16_(CaCp)_2_]^−^ triple-decked complex was a minimum on the potential energy surface with high electronic stability. All the B–B and Co–B bond lengths were found to be almost the same as in isomers **I** and **II** of CoB_16_^−^ (Supplementary Table 3). We have further performed AdNDP analyses and found that the triple-decked sandwich complex exhibits exactly the same chemical bonding pattern as the parent CoB_16_^−^ (Supplementary Figs 6–8). The Natural Population Analysis (NPA) charge on Ca was found to be +1.54, consistent with the initial hypothesis and the charge-transfer nature of the triple-decked [CoB_16_(CaCp)_2_]^−^ sandwich complex. Thus, the CoB_16_^−^ molecular drum can serve as a building block for the design of novel cluster-assembled nanomaterials. The high stability of the CoB_16_^−^ drum structures may also help the search for new metal-boride phases containing various boron ring units^43^.

We have produced and characterized a large Co-doped boron cluster, CoB_16_^−^, using photoelectron spectroscopy and quantum-chemical calculations. Extensive computational searches established two high symmetry (D_8d_ and C_4v_) drum-like structures with Co sandwiched by two B_8_ rings as nearly degenerate global minima. The CoB_16_^−^ molecular drums represent the highest coordination for a metal atom known in chemistry and opens new possibilities for designing novel boron-based nanomaterials. First, the CoB_16_^−^ drums may be considered as the embryo to make filled boron nanotubes due to the significant B-B bonding between the two B_8_ rings. Second, there are possibilities to observe larger doped-boron clusters with even higher coordination number to further push the limit of coordination number in chemistry. Third, we have demonstrated one possibility to use CoB_16_^−^ as a building block of new cluster-assembled nanomaterials in a triple-decked complex.

## Methods

### Experimental methods

The experiment was carried out using a magnetic-bottle PES apparatus equipped with a laser vaporization cluster source^44^. Briefly, the CoB_16_^−^ anion clusters were produced by laser vaporization of a cold-pressed target composed of Co and isotopically enriched ^11^B. Bismuth was added as a binder and it also provided a convenient calibrant (Bi^−^) for the PES experiment. Clusters formed in the nozzle were entrained in a He carrier gas and underwent a supersonic expansion to form a collimated cluster beam. The He carrier gas was seeded with 5% Ar for better cooling of the entrained clusters^22^. The anionic clusters were extracted from the collimated cluster beam and analysed by a time-of-flight mass spectrometer. The CoB_16_^−^ anion clusters were mass selected and decelerated before being photodetached by a laser beam at 193 nm (6.424 eV) from an ArF excimer laser or 266 nm (4.661 eV) from a Nd:YAG laser. Photoelectrons were collected at nearly 100% efficiency by a magnetic bottle and analysed in a 3.5 m long flight tube. The resolution of the apparatus, ΔEk/Ek, was about than 2.5%, that is, ∼25 meV for 1 eV electrons.

### Theoretical methods

Search for the global minimum of CoB_16_^−^ was performed using the Coalescence Kick program^45^ at the PBE0/3-21G level of theory^46^,^47^. The Coalescence Kick algorithm generated ∼10,000 trial structures for each spin multiplicity (singlet, triplet and quintet), followed by geometry optimization. Low-lying isomers within 25 kcal mol^−1^ were further refined at a more expansive basis set, Def2-TZVP^48^. For each structure, vibrational frequencies were calculated and imaginary frequencies were followed to ensure that the isomer corresponded to a true minimum on the potential energy surface. Spin contamination was found to be <10% in all DFT calculations. For selected isomers, we performed additional geometry optimization at various DFT levels, as well as more accurate single-point coupled-cluster calculations [ROCCSD(T)/6-311+G(2df)], to reliably establish the relative energy ordering. Vertical detachment energies of the three lowest energy structures were calculated at three different methods (UPBE0, UB3LYP and ROCCSD(T)) to compare with the experimental data. The VDEs were obtained as the difference in energy between the ground state of the anion and selected low-lying electronic states of the neutral molecule at the geometry of the anion. All calculations were done using GAUSSIAN-09 (ref. 49).

To understand the chemical bonding, we carried out electron localization analyses using the AdNDP method^38^ at the PBE0/6-31G(d) level of theory. Previously, AdNDP results have been shown to be insensitive to the level of theory or basis set used^50^. The AdNDP analysis is based on the concept of electron pairs as the main elements of chemical bonds. It represents the molecular electronic structure in terms of *n*-centre two-electron (*n*c–2e) bonds, recovering the familiar lone pairs (1c–2e) and localized 2c–2e bonds or delocalized *n*c–2e bonds (3≤*n*≤total number of atoms in the system). The MOLEKEL 5.4.0.8 program^51^ is used for molecular structure and AdNDP bond visualizations.

## Additional information

**How to cite this article:** Popov, I. A. *et al*. Cobalt-centred boron molecular drums with the highest coordination number in the CoB_16_^−^ cluster. *Nat. Commun.* 6:8654 doi: 10.1038/ncomms9654 (2015).

## Supplementary Material

Supplementary Information Supplementary Figures 1-8 and Supplementary Tables 1-3

## Figures and Tables

**Table 1:**
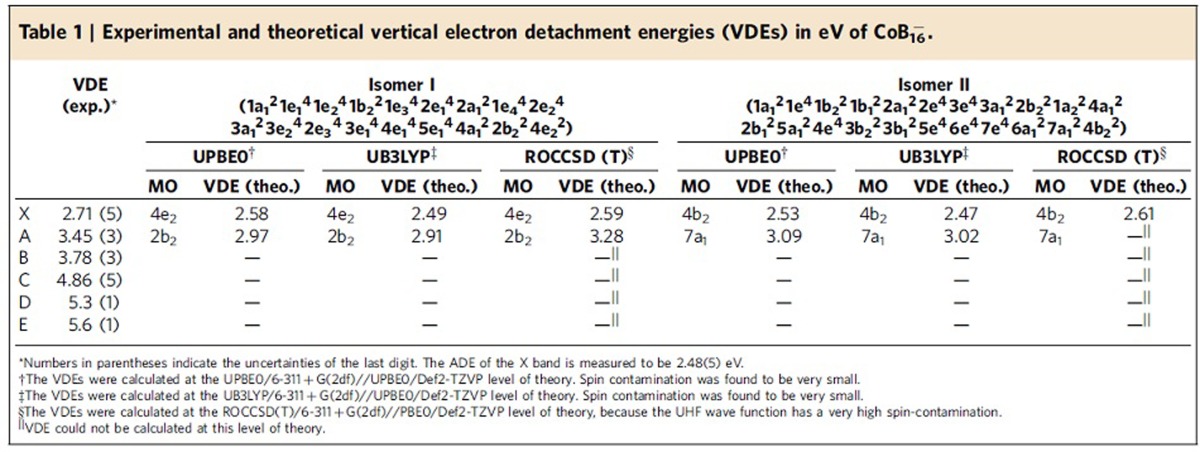
Experimental and theoretical vertical electron detachment energies (VDEs) in eV of CoB_16_^−^.
