# Supplementary material for: Cobalt-centred boron molecular drums with the highest coordination number in the CoB16− cluster
Source: Nat Commun. 2015 Oct 12;6:8654. doi: 10.1038/ncomms9654 (PMC4633964; doi:10.1038/ncomms9654)
Supplement: Supplementary Information — Supplementary Figures 1-8 and Supplementary Tables 1-3 [file ncomms9654-s1.pdf]

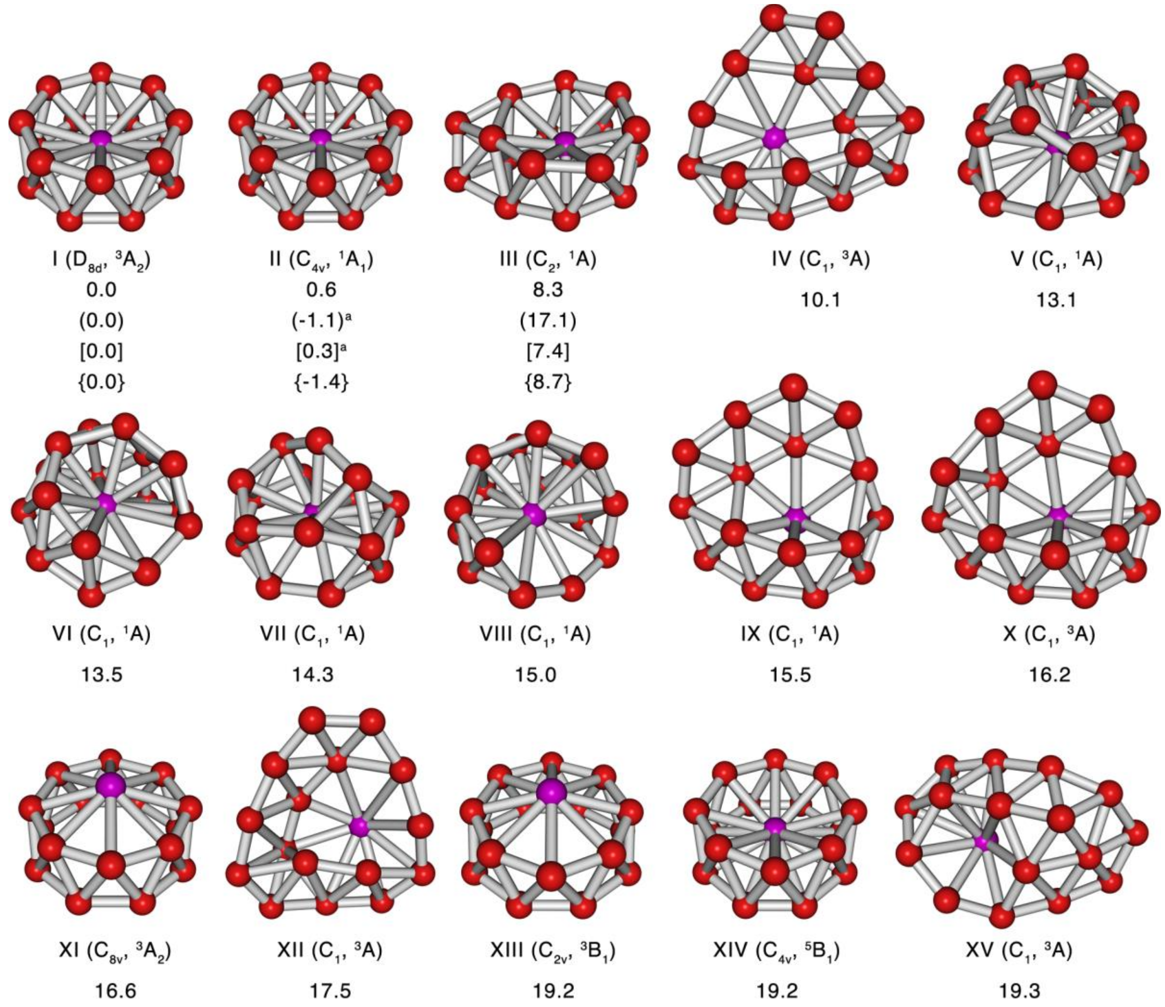

Supplementary Figure 1. Relative energies (in kcal/mol) of the lowest lying (<20 kcal/mol above the global minimum) isomers of CoB_{16}^- at the PBE0/Def2-TZVP level of theory. B3LYP/Def2-TZVP, TPSSh/Def2-TZVP, and ROCCSD(T)/6-311+G(2df)//PBE0/Def2-TZVP relative energies for the three lowest energy isomers **I, II,** and **III** are shown in parenthesis, square brackets, and curly brackets, respectively. All energies are corrected for zero-point energies (ZPE) at their respective levels of theory aside from single-point coupled-cluster calculations, which are ZPE corrected at PBE0/Def2-TZVP. The C_{4V} structure (isomer **II**) has one imaginary frequency at this level of theory. Geometry optimization following the imaginary frequency leads to the local minimum structure possessing C_4 symmetry. However, including ZPE corrections the vibrationally averaged structure has effectively C_{4V} symmetry.

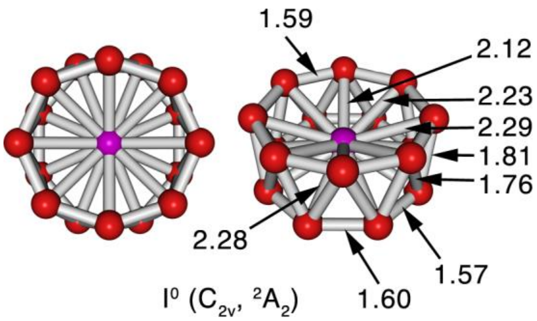

Supplementary Figure 2. Two views of the neutral CoB_{16} isomer I^0.

Its point group symmetries and spectroscopic states are shown in parenthesis. All distances are in Å.

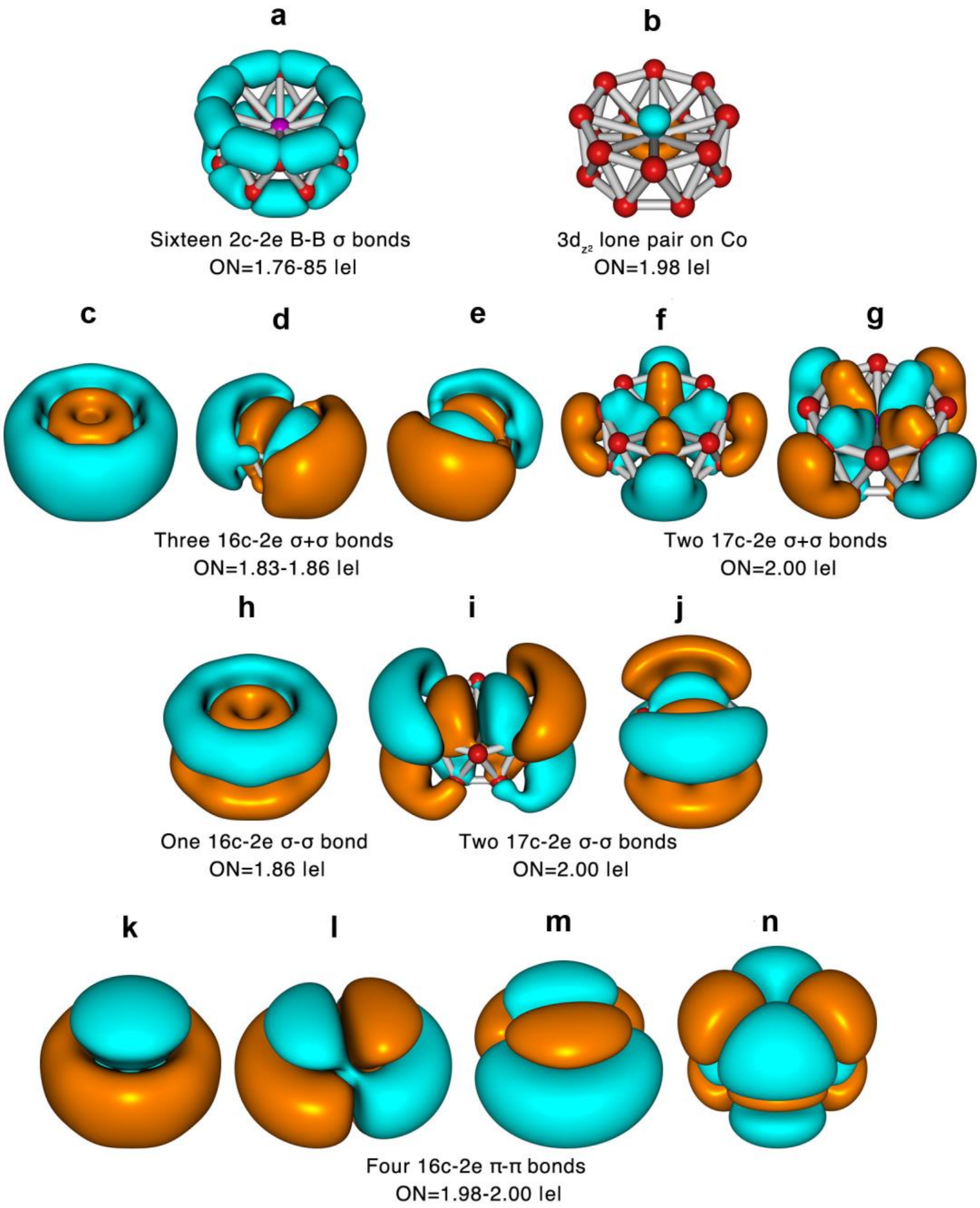

Supplementary Figure 3. The AdNDP chemical bonding picture obtained for isomer II (C_{4v}, $^1A_1$) of CoB_{16}^-.

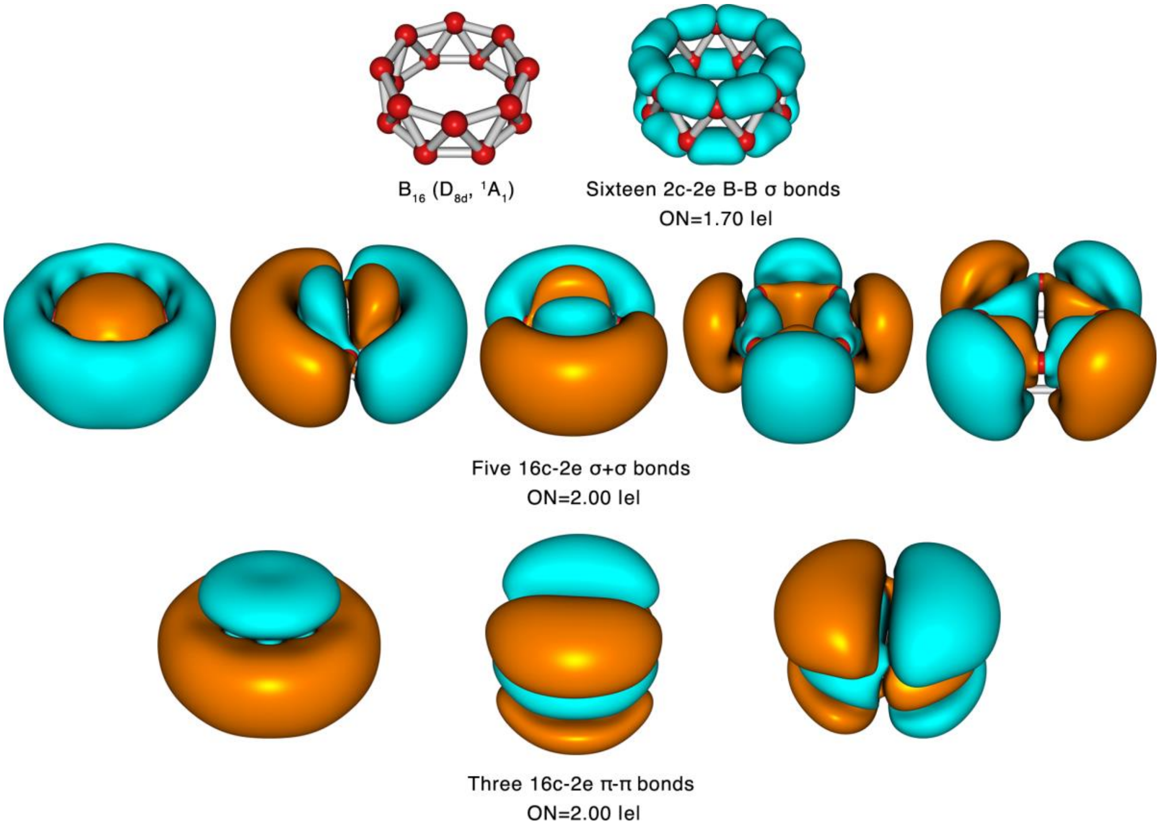

Supplementary Figure 4. The AdNDP chemical bonding picture obtained for the tubular isomer of the B_{16} cluster.

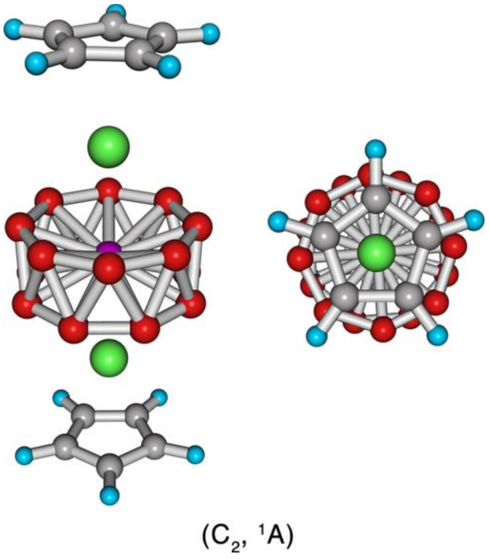

Supplementary Figure 5. Two views of the triple-decked $[CoB_{16}(CaCp)_2]^-$ cluster. Its point group symmetry and spectroscopic state are shown in parenthesis. Color scheme: Ca is green, C is grey, H is blue, B is red, and Co is magenta.

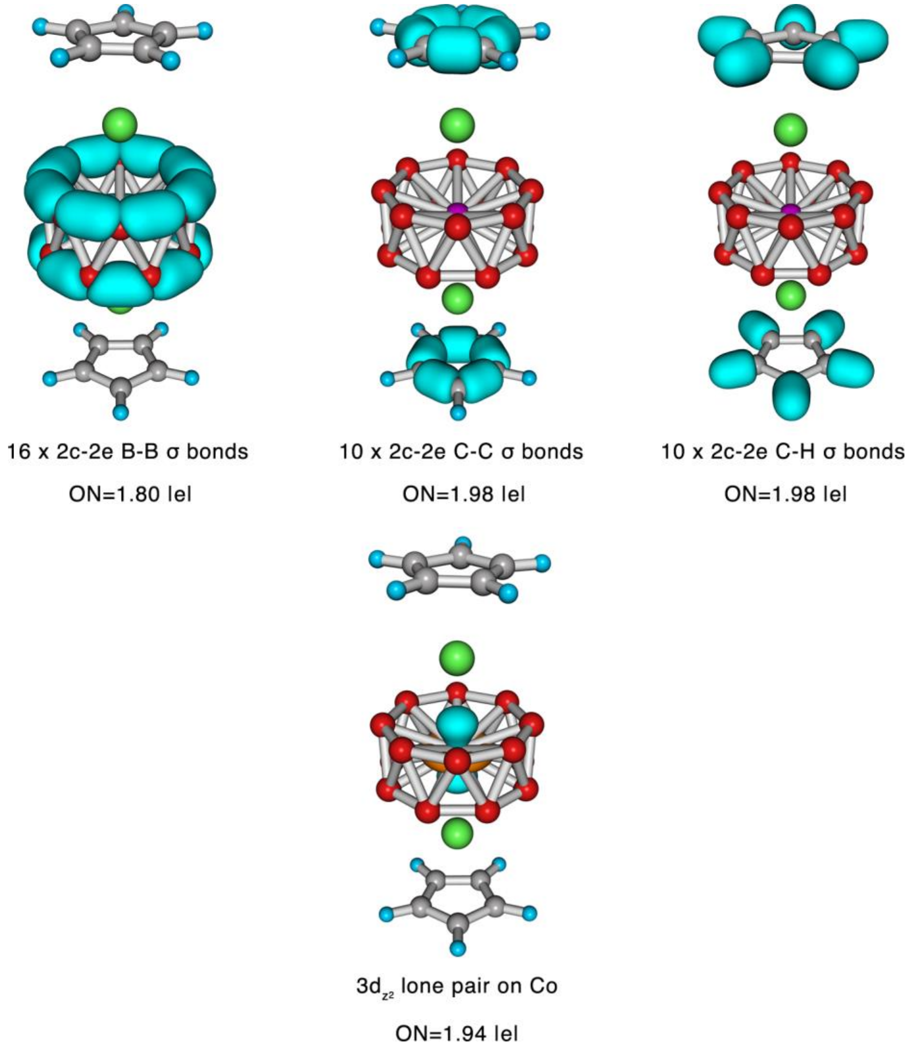

Supplementary Figure 6. Localized elements of chemical bonding: 1c-2e (lone pairs) and 2c-2e (classical two-center two-electron) bonds found on both the CoB_{16}^{3-} and $C_5H_5^{-}$ fragments.

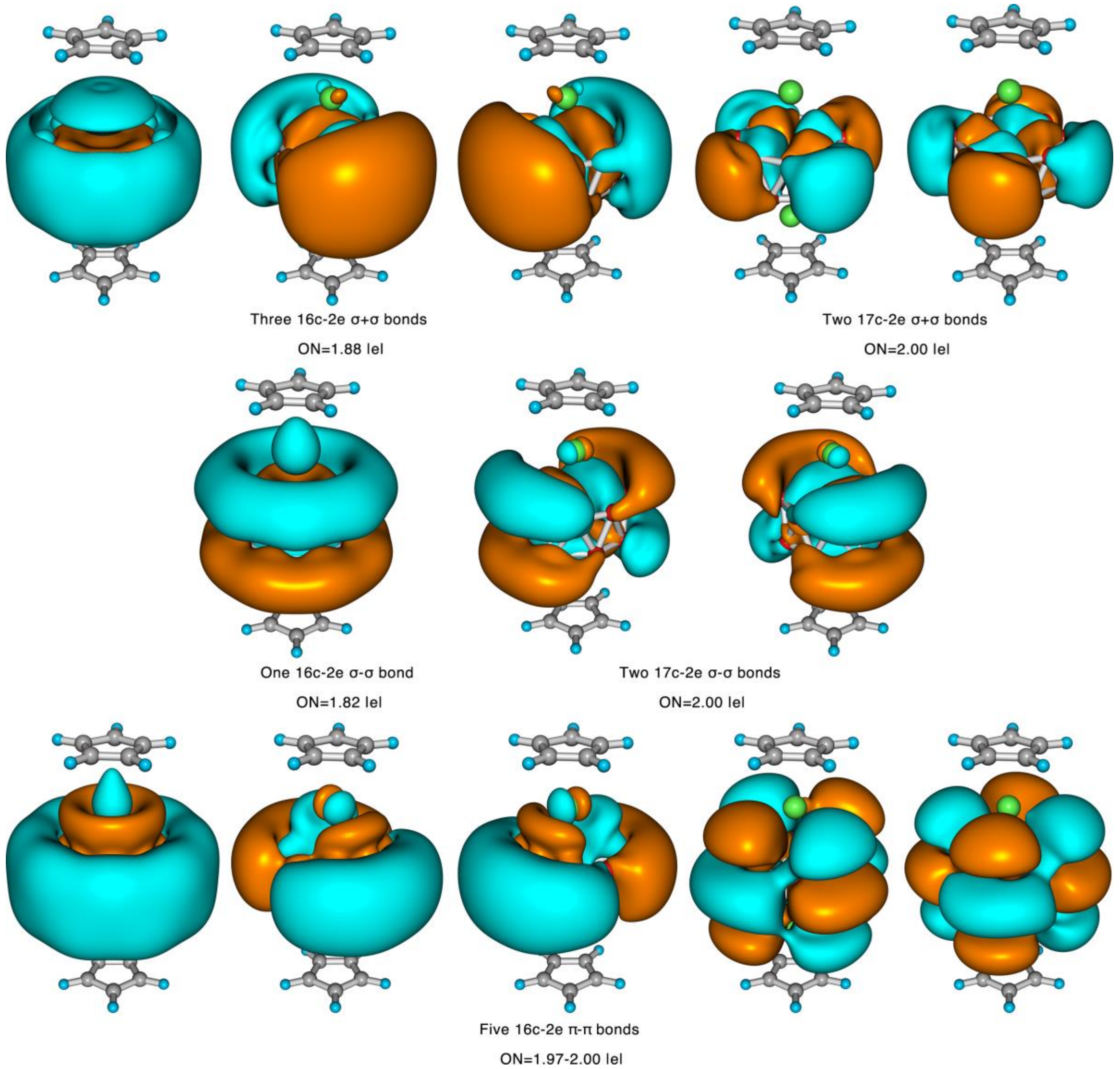

Supplementary Figure 7. Delocalized elements of chemical bonding found on the CoB_{16}^{3-} fragment in $[CoB_{16}(CaCp)_2]^{-}$.

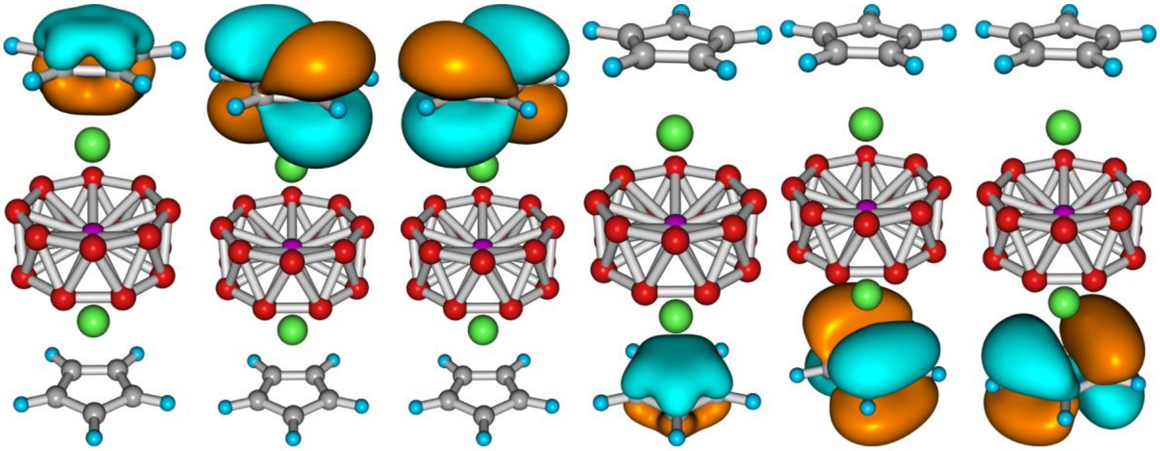

Supplementary Figure 8. Delocalized elements of chemical bonding found on the $C_5H_5^-$ fragments in $[CoB_{16}(CaCp)_2]^-$.

Supplementary Table 1.
Cartesian coordinates of the three lowest energy isomers of the CoB_{16}^{-} cluster (I-III), neutral CoB_{16} isomer I^{0}, and the triple-decked $[CoB_{16}(CaCp)_2]^{-}$ sandwich compound at the PBE0/Def2-TZVP level of theory.

**I**	5 0.000000000 2.072443000 0.806679000
	27 0.000000000 0.000000000 0.000000000
	5 -1.465438000 -1.465438000 0.806679000
	5 1.465438000 -1.465438000 0.806679000
	5 2.072443000 0.000000000 0.806679000
	5 -2.072443000 0.000000000 0.806679000
	5 -1.465438000 1.465438000 0.806679000
	5 1.465438000 1.465438000 0.806679000
	5 0.000000000 -2.072443000 0.806679000
	5 -1.914687000 0.793089000 -0.806679000
	5 0.793089000 1.914687000 -0.806679000
	5 0.793089000 -1.914687000 -0.806679000
	5 1.914687000 -0.793089000 -0.806679000
	5 -0.793089000 1.914687000 -0.806679000
	5 1.914687000 0.793089000 -0.806679000
	5 -0.793089000 -1.914687000 -0.806679000
	5 -1.914687000 -0.793089000 -0.806679000
**II**	5 -1.926102000 0.772790000 -0.817317000
	27 0.000000000 0.000000000 -0.003007000
	5 1.926102000 0.772790000 -0.817317000
	5 0.772790000 -1.926102000 -0.817317000
	5 -0.772790000 -1.926102000 -0.817317000
	5 0.772790000 1.926102000 -0.817317000
	5 -0.772790000 1.926102000 -0.817317000
	5 -1.926102000 -0.772790000 -0.817317000
	5 1.926102000 -0.772790000 -0.817317000
	5 0.000000000 2.103180000 0.773458000
	5 -2.103180000 0.000000000 0.773458000
	5 1.423652000 -1.423652000 0.865236000
	5 0.000000000 -2.103180000 0.773458000
	5 -1.423652000 1.423652000 0.865236000
	5 -1.423652000 -1.423652000 0.865236000
	5 2.103180000 0.000000000 0.773458000
	5 1.423652000 1.423652000 0.865236000

**III**	27 0.000000000 0.000000000 0.254632000<br>5 -1.598686000 -0.027003000 -1.298828000<br>5 1.376226000 0.157703000 1.718441000<br>5 -0.769403000 -0.124147000 -2.723566000<br>5 -0.095219000 1.023694000 -1.533573000<br>5 -1.376226000 -0.157703000 1.718441000<br>5 0.000000000 -0.906301000 2.116848000<br>5 0.769403000 0.124147000 -2.723566000<br>5 1.063171000 -1.616745000 -0.411190000<br>5 -1.063171000 1.616745000 -0.411190000<br>5 -1.181627000 1.489917000 1.183241000<br>5 2.033584000 -0.232101000 0.261119000<br>5 1.598686000 0.027003000 -1.298828000<br>5 1.181627000 -1.489917000 1.183241000<br>5 -2.033584000 0.232101000 0.261119000<br>5 0.095219000 -1.023694000 -1.533573000<br>5 0.000000000 0.906301000 2.116848000
**I^0**	5 1.448199000 1.506481000 -0.788339000<br>27 0.000000000 0.000000000 0.000385000<br>5 0.000000000 -2.162238000 -0.768730000<br>5 -1.949398000 0.000000000 -0.836223000<br>5 -1.448199000 1.506481000 -0.788339000<br>5 1.448199000 -1.506481000 -0.788339000<br>5 1.949398000 0.000000000 -0.836223000<br>5 0.000000000 2.162238000 -0.768730000<br>5 -1.448199000 -1.506481000 -0.788339000<br>5 1.990339000 -0.802304000 0.769780000<br>5 0.801384000 1.832645000 0.820516000<br>5 -1.990339000 -0.802304000 0.769780000<br>5 -1.990339000 0.802304000 0.769780000<br>5 1.990339000 0.802304000 0.769780000<br>5 -0.801384000 1.832645000 0.820516000<br>5 -0.801384000 -1.832645000 0.820516000<br>5 0.801384000 -1.832645000 0.820516000
**$[CoB_{16}(CaCp)_2]^-$**	5 0.407037000 -0.804126000 -2.040487000<br>27 0.000000000 0.000000000 0.006130000<br>5 1.158948000 -0.803673000 1.740415000<br>5 -1.734320000 -0.803739000 1.164948000<br>5 -2.046524000 -0.804227000 -0.400973000<br>5 2.046585000 -0.804103000 0.413103000<br>5 1.734263000 -0.803804000 -1.152792000<br>5 -1.158844000 -0.803729000 -1.728218000<br>5 -0.407004000 -0.804265000 2.052622000<br>5 2.046524000 0.804227000 -0.400973000<br>5 -0.407037000 0.804126000 -2.040487000<br>5 -1.158948000 0.803673000 1.740415000

	5	-2.046585000	0.804103000	0.413103000
	5	1.158844000	0.803729000	-1.728218000
	5	-1.734263000	0.803804000	-1.152792000
	5	0.407004000	0.804265000	2.052622000
	5	1.734320000	0.803739000	1.164948000
	20	0.000000000	2.821886000	0.007190000
	20	0.000000000	-2.821886000	0.007190000
	6	0.330494000	5.286924000	1.144656000
	6	1.201179000	5.277963000	0.026871000
	6	0.407202000	5.266431000	-1.146524000
	6	-0.954154000	5.268140000	-0.753962000
	6	-1.001663000	5.280773000	0.661969000
	6	0.954154000	-5.268140000	-0.753962000
	6	1.001663000	-5.280773000	0.661969000
	6	-0.407202000	-5.266431000	-1.146524000
	6	-1.201179000	-5.277963000	0.026871000
	6	-0.330494000	-5.286924000	1.144656000
	1	2.285178000	5.298079000	0.062981000
	1	0.631016000	5.315278000	2.186602000
	1	-1.900047000	5.303272000	1.269566000
	1	-1.809892000	5.277818000	-1.420519000
	1	0.776775000	5.274625000	-2.166339000
	1	-0.631016000	-5.315278000	2.186602000
	1	-2.285178000	-5.298079000	0.062981000
	1	-0.776775000	-5.274625000	-2.166339000
	1	1.809892000	-5.277818000	-1.420519000
	1	1.900047000	-5.303272000	1.269566000

Supplementary Table 2.

The first two calculated VDE values for isomer III (C_2, ^1A) of CoB_{16}^- at three levels of theory.

Isomer **III** ($1a^2 2a^2 1b^2 3a^2 2b^2 4a^2 3b^2 4b^2 5a^2 5b^2 6a^2 6b^2 7a^2 7b^2 8a^2 9a^2 10a^2 8b^2 11a^2 9b^2$ $12a^2 10b^2 13a^2 14a^2 11b^2 12b^2 15a^2 13b^2 16a^2$)					
UPBE0[a]		UB3LYP[b]		ROCCSD(T)[c]	
MO	VDE	MO	VDE	MO	VDE
16a	3.35	16a	3.32	16a	3.65
13b	3.62	13b	3.53	13b	—[d]

[a] The VDEs were calculated at the UPBE0/6-311+G(2df)//UPBE0/Def2-TZVP level of theory. Spin-contamination was found to be very small.

[b] The VDEs were calculated at the UB3LYP/6-311+G(2df)//UPBE0/Def2-TZVP level of theory. Spin-contamination was found to be very small.

[c] The VDEs were calculated at the ROCCSD(T)/6-311+G(2df)//PBE0/Def2-TZVP level of theory, because the UHF wave function has a very high spin-contamination.

[d] VDE could not be calculated at this level of theory.

Supplementary Table 3.

Comparison of the bond distances in B_{16}, CoB_{16}^- (isomers I and II), and $[CoB_{16}(CaCp)_2]^-$ clusters at the PBE0/Def2-TZVP level of theory. All values are in Å.

	B_{16}	CoB_{16} (structure **I^0**)	CoB_{16}^- (isomer **I**)	CoB_{16}^- (isomer **II**)	$[CoB_{16}(CaCp)_2]^-$
R(Co-B)	-	2.12-2.29	2.22	2.19-2.24	2.24
R(peripheral B-B)	1.63	1.57-1.60	1.59	1.55-1.63	1.60
R(B-B between the rings)	1.64	1.76-1.81	1.80	1.78-1.87	1.80
